# miR-224-5p Attenuates Allergic Responses in Mice with Allergic Rhinitis by Modulating the Th1/Th2 Response

**DOI:** 10.1155/2024/5531970

**Published:** 2024-02-29

**Authors:** Yuxiao Li, Ran An, Mingjin Wu, Jiayan He, Xiaoguang He

**Affiliations:** ^1^Department of Otorhinolaryngology Head and Neck Surgery, First Affiliated Hospital of Kunming Medical University, Kunming, Yunnan 650032, China; ^2^Department of Otorhinolaryngology, The Seventh Affiliated Hospital Sun-Yat Sen University, Shenzhen, Guangdong 517108, China; ^3^Department of Otorhinolaryngology, Yuhang Second People's Hospital, Hangzhou, Zhejiang 311100, China; ^4^Central Supply Department, First Affiliated Hospital of Kunming Medical University, Kunming, Yunnan 650032, China

## Abstract

**Background:**

Allergic rhinitis (AR) is a common chronic respiratory disease that has become a global health problem. miRNAs play an important role in multiple immune and inflammatory diseases, including AR. In this work, the mechanism by which miR-224-5p regulates AR in vivo and in vitro was examined.

**Methods:**

Human nasal epithelial cells (HNEpCs) were used to establish an AR cell model induced by Der P1, and C57BL/6 mice were used to establish an AR animal model induced by OVA (ovalbumin). RT-qPCR was used to determine the level of miR-224-5p; western blot analysis was used to determine GATA3; ELISA was used to determine the levels of OVA-specific IgE, IFN-*γ*, IL-4, IL-5, and IL-13; flow cytometry was used to determine the differentiation of Th1 and Th2 cells; and HE and PAS staining was used to observe the histopathological alterations in the mouse nasal mucosa and spleen.

**Results:**

miR-224-5p was downregulated in nasal mucosa from mice with AR and an AR cell model. Overexpressed miR-224-5p can improve AR development and attenuate AR symptoms by regulating GATA3-mediated Th1/Th2 responses.

**Conclusion:**

miR-224-5p attenuates allergic reactions in mice with AR by regulating the Th1/Th2 response.

## 1. Introduction

Allergic rhinitis (AR) is a common chronic respiratory disease mediated by immunoglobulin E (IgE), which is characterized by nasal clogging, nasal discharge, sneezing, nasal friction, and nasal pruritus [1]. AR is a worldwide health problem affecting approximately 40% of the total population worldwide [2]. Current AR therapies incorporate allergen avoidance, medication, and immunotherapy [3]. Nevertheless, treatment failure or unsatisfactory results may still occur with these regimens [4]. Therefore, it is important to explore AR etiopathogenesis, find novel remedial targets, and develop new therapeutic drugs.

The imbalance of T helper type 1 (Th1) cells and T helper type 2 (Th2) cells was proposed as a major inducer of IgE-mediated allergic inflammation [5–7]. A previous study reported that the fundamental cause of allergic diseases may be the mutual effect of genetic and ambient factors [8]. When the body is exposed to a certain concentration of allergen in the environment for a long time, antigen-presenting cells submit the allergen to CD4 T lymphocytes, and CD4 T cells liberate cytokines to provoke B cells to differentiate into plasma cells, which ultimately promotes the generation of IgE and restrains the Th1 reaction [9]. It causes the release of inflammatory cytokines that induce a Th2 response when IgE antibodies combine with receptors on mast cells and eosinophils [10]. AR manifests as increased Th2 cells and decreased Th1 cells [11]. The imbalance in CD4 T-cell subsets and the accumulation of eosinophils and mast cells in the nasal mucosa are important features of AR. Therefore, normalizing the Th1/Th2 ratio and inhibiting the accumulation of eosinophils and mast cells are key for AR treatment.

MicroRNAs (miRNAs) are one of the fundamental regulatory mechanisms of gene expression and bind complementary sequences of the 3′UTR on target mRNA transcripts to control posttranscriptional gene expression [12]. Studies have shown that miRNAs play an important role in many immune and inflammatory diseases, including AR [13, 14]. miRNAs accelerate the evolution of allergic diseases by influencing the Th1/Th2 balance, facilitating inflammatory responses in the epithelium and tissues, and activating innate immune cells. miR-125a, miR-21, and miR-140 have been demonstrated to be related to symptom severity and Th1/Th2 imbalance in AR [15, 16]. Recent studies have shown that miR-224-5p plays an important role in AR progression [17]. Nevertheless, the latent mechanism of miR-224-5p in AR evolution remains unclear.

GATA3, a key regulator of Th2 cells, has been reported to be intimately linked to Th2 proliferation and differentiation [18]. GATA3 is a target gene of miRNA and is involved in regulating immune cell proliferation and differentiation and in regulating congenital and accommodative immune responses [18–20]. For example, miR-466a-3p attenuates AR in mice by targeting GATA3 [21]. miR-135a regulates eosinophils and mast cells and allergic inflammation in AR by targeting GATA3 [22]. Notably, it was predicted that miR-224-5p could directly target GATA3 through the starBase website (https://starbase.sysu.edu.cn/), so we conjectured that miR-224-5p might control the generation and progression of AR by GATA3.

The aim of this study was to identify the mechanism by which miR-224-5p attenuates the allergic response in mice with AR by targeting GATA3 to regulate the Th1/Th2 response and provide a reference for the development of effective and novel remedies for AR.

## 2. Materials and Methods

### 2.1. Cell Culture

Human nasal epithelial cells (HNEpCs) were purchased from Huatuo Biology (Shenzhen, China). HNEpCs were cultured in human airway epithelial cell growth medium (Procell, Wuhan, China). CD4 T cells were purchased from IPHASE Biotechnology Co., Ltd. (isolated from normal human peripheral blood; the purity of the cells was ≥85%) and cultured in RPMI-1640 complete substrate (Gibco, USA). All cell culture media contained 1% penicillin/streptomycin (Gibco, USA). RPMI-1640 complete substrate contained 10% fetal serum. The incubator conditions were 37°C and 5% CO_2_. Microscopic observation of cell morphology, routine medium change, and passaging were performed.

### 2.2. Establishment of the AR Cell Model

An AR cell model was established by treating HNEpC cells with 1 *μ*g/mL Der P1 (Sigma, USA) for 24 hr [23]. The treated HNEpCs were cocultured with CD4 T cells, flow cytometry was performed to observe the differentiation of Th1 and Th2 cells, and the levels of Th1/Th2-related cytokines were analyzed by ELISA.

### 2.3. Establishment of the AR Mouse Model

Female C57BL/6 (18–20 g, 6–8 weeks old) mice were purchased from the Animal Experimental Center of Kunming Medical University. The AR mouse model was generated by ovalbumin (OVA, Sigma, USA) induction, and 100 *μ*L of saline containing 50 *μ*g of OVA and 2 mg of Al(OH)_3_ was injected intraperitoneally on days 0, 7, and 14. Then, 20 *μ*L of saline containing 400 *μ*g OVA was directly injected intranasally every 4 days from days 21 to 28. After 28 days, nasal mucosa and spleen tissues were collected for analysis after sacrifice [24, 25].

The miR-224-5p mimic group mice were intraperitoneally sensitized and intranasally challenged with OVA, similar to that of the AR group. On days 18, 21, 24, and 27, the mimic group was treated intranasally with 20 *μ*L saline containing 50 *μ*g miR-224-5p mimic to intervene with the incidence of AR. miR-224-5p mimic was provided by Sangon Biotech (Shanghai, China).

### 2.4. Assessment of Allergy Symptoms

The number of mice sneezing and with nasal rubbing was counted, and the frequency within 15 min was calculated after the last OVA challenge. The average of the observations was used in the final results to assess the AR symptoms of mice.

### 2.5. RT-qPCR

Total RNA was extracted from the AR mouse nasal mucosa and spleen and the AR cell model by TRIzol Reagent (Beyotime, China). Then, the RNA quality and concentration were measured. Transcribed RNA was reverse transcribed into cDNA. RT-qPCR was performed with U6 as an internal control. The detailed primer sequences are shown in Table 1, and differential gene expression was analyzed by the 2^−*ΔΔ*Ct^ method.

### 2.6. Western Blot

Proteins were isolated from cells or nasal mucosa with RIPA buffer containing 1% protease inhibitors. Protein concentrations were determined according to the specifications of the bicinchoninic acid assay (BCA, Solarbio, China) kit. 50 *µ*g of protein was loaded per lane, and total proteins were separated by SDS-PAGE (5% concentrated gel, 10% separating gel), transferred to PVDF membranes (Millipore, USA) and then blocked with 5% nonfat milk powder for 1.5 hr at room temperature. The following diluted primary antibodies were added and incubated overnight at 4°C: anti-*β*-actin (1 : 1,000, ab8226 Abcam, UK) and anti-GATA3 (ab106625, 1 : 1,000, Abcam, UK). Next, the membranes were incubated with secondary antibodies (1 : 4,000, ab97051) for 1 hr at room temperature and developed with an ECL kit. Finally, the bands were semiquantitatively analyzed by ImageJ software.

### 2.7. Flow Cytometry

Cells were harvested and suspended in medium with 10% FBS. The designated cells were collected and suspended after culturing for 72 hr. The cell suspensions were treated with PMA (25 ng/mL), ionomycin (1 *μ*g/mL), and Golgistop (5 ng/mL) for 4 hr at 37°C. Th1 and Th2 cell subsets were analyzed by the intracellular cytokine IFN-*γ* and IL-4 (CD3^+^CD4^+^).

### 2.8. ELISA

The corresponding ELISA kits were used according to the protocol (Solarbio, China) to determine the levels of IFN-*γ*, IL-4, IL-5, and IL-13 in the supernatants of treated HNEpCs cocultured with CD4 T cells and the levels of OVA-specific IgE, IFN-*γ*, IL-4, IL-5, and IL-13 in serum from mice.

### 2.9. Dual-Luciferase Gene Analysis

The wild-type (WT) or mutant (MUT) 3′-UTR sequence of GATA3 was inserted into the pGL3 promoter vector. GATA3 WT or GATA3 MUT and miR-224-5p control or miR-224-5p mimic were transfected into HEK-293T cells. A dual-luciferase reporter assay system was used to assess luciferase activity.

### 2.10. Histological Analysis

Nasal mucosa and spleen tissues were isolated from mice and immobilized with 4% paraformaldehyde for 24 hr at room temperature. The dehydrated tissue specimens were paraffin-embedded and cut into 4–5 *μ*m sections. Pathological changes in the nasal mucosa and spleen were observed by HE and PAS staining.

### 2.11. Statistical Analysis

The analytical results are expressed as the mean ± SD. All statistical analyses were performed with GraphPad Prism 8.0. All cell experiments were performed with three parallel groups and repeated three times. All animal experiments were performed with five parallel groups and repeated three times. One-way ANOVA and *T* test were used for the analysis by Tukey's test post hoc tests. The data were statistically significant if *P* < 0.05.

## 3. Results

### 3.1. Downregulation of miR-224-5p in AR Mouse and Cell Models

**Figure 1:**
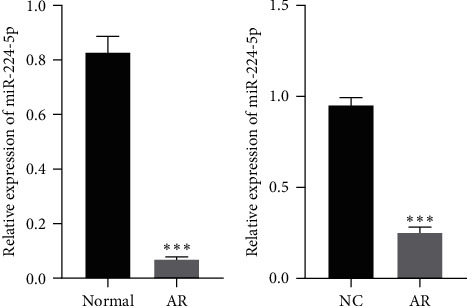
Downregulation of miR-224-5p in mice with AR and a cell model: (a) RT-qPCR for testing the miR-224-5p level in the nasal mucosa of normal control mice and mice with AR and (b) RT-qPCR for testing the miR-224-5p level in normal control and AR cell models.  ^*∗∗∗*^*P* < 0.001.

In the OVA-induced mouse AR model, miR-224-5p expression was observably reduced, as shown by RT-qPCR (Figure 1(a)). In the AR cell model induced by Der P1, the miR-224-5p level was observably reduced, as shown by RT-qPCR (Figure 1(b)).

### 3.2. Overexpression of miR-224-5p Attenuates AR Development

**Figure 2:**
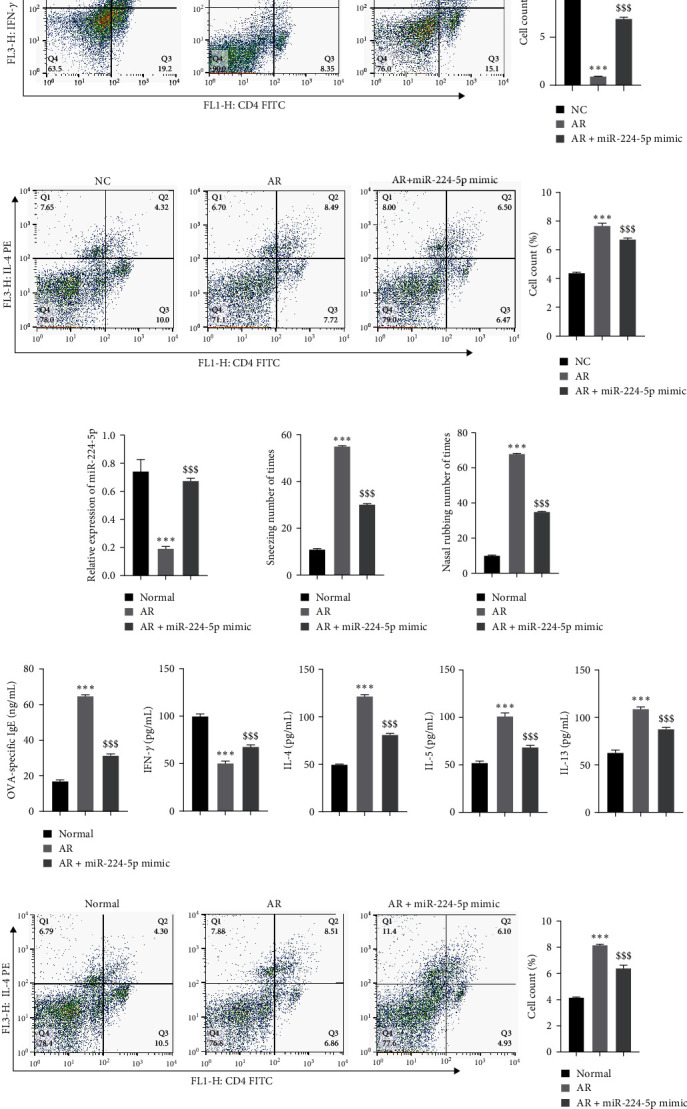
Overexpression of miR-224-5p attenuates the AR process: (a) RT-qPCR for measuring miR-224-5p transfection efficiency, (b) ELISA for measuring the levels of IFN-*γ*, IL-4, IL-5, and IL-13, (c) flow cytometry for detecting Th1 differentiation, (d) flow cytometry for detecting Th2 differentiation, (e) RT-qPCR for testing the miR-224-5p level, (f) observation of the frequency of sneezing, (g) observation of the frequency of nasal friction, (h) ELISA for assessing the levels of OVA-specific IgE, IFN-*γ*, IL-4, IL-5, and IL-13, (i) flow cytometry for detecting Th2 differentiation in mouse spleen, (j) HE staining for observing histopathological alterations of mouse nasal mucosa (scale bar = 100 *µ*m), and (k) PAS staining for observing the histopathological alterations of nasal mucosa (scale bar = 100 *µ*m).  ^*∗∗*^*P* < 0.01,  ^*∗∗∗*^*P* < 0.001 vs. the NC group or normal group; ^$$$^*P* < 0.001 vs. the AR group.

To assess the influence of miR-224-5p on AR development and progression, miR-224-5p was overexpressed in the CD4 T cells and cocultured with AR cell model, and its effect on CD4 T differentiation was observed. First, RT-qPCR showed successful transfection of miR-224-5p (Figure 2(a)). Compared to the NC group, IFN-*γ* was observably decreased, and IL-4, IL-5, and IL-13 were observably elevated in the supernatant of the AR cells cocultured with CD4 T cells. Compared to the AR group, IFN-*γ* was prominent, while the expression of IL-4, IL-5, and IL-13 was observably decreased after the addition of the miR-224-5p mimic (Figure 2(b)). Flow cytometry showed that compared to the NC group, the amount of Th1 cells was observably decreased, and the amount of Th2 cells was observably increased in the AR group. The Th1/Th2 balance was reversed after overexpression of miR-224-5p (Figures 2(c) and 2(d)). Next, the influence of miR-224-5p overexpression was assessed in an AR mouse model. RT-qPCR showed that miR-224-5p expression was observably upregulated in mice after overexpression of miR-224-5p (Figure 2(e)). The results showed that compared to the normal group, the frequency of sneezing and nasal friction in the mice with AR was observably elevated, while the frequency of snoring and nasal friction in the mice with AR was decreased after miR-224-5p mimic transfection (Figures 2(f) and 2(g)). ELISA showed that compared to the normal group, the levels of OVA-specific IgE, IL-4, IL-5, and IL-13 were observably elevated, while the content of IFN-*γ* was observably decreased, and the opposite results were observed after the addition of the miR-224-5p mimic (Figure 2(h)). A prominent increase in the differentiation of Th2 cells in AR mouse spleens was detected by flow cytometry, and this change decreased after miR-224-5p administration (Figure 2(i)). HE and PAS staining showed abundant eosinophil infiltration and goblet cell proliferation in the AR mouse nasal mucosa compared to the normal group, while eosinophil infiltration and goblet cell proliferation were prominently decreased after miR-224-5p administration (Figures 2(j) and 2(k)). The above results suggested that overexpressed miR-224-5p can moderate allergic symptoms and lessen Th2 cell differentiation and inflammatory cytokine levels in AR.

### 3.3. Targeted Regulation of GATA3 by miR-224-5p

**Figure 3:**
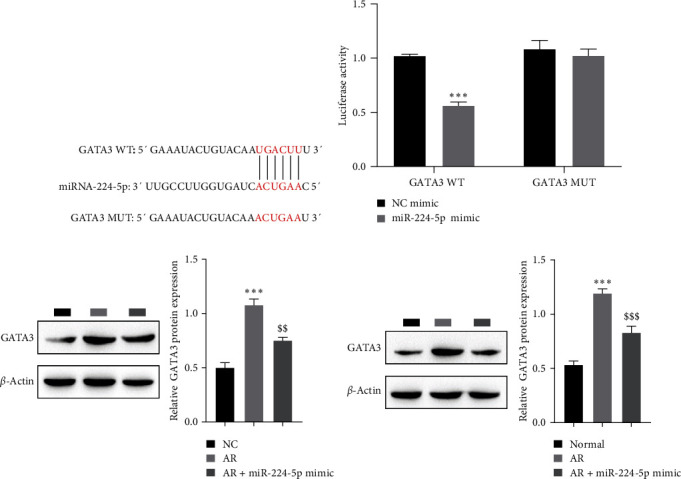
miR-224-5p targets GATA3: (a) starBase predicted the target binding site of miR-224-5p and GATA3, (b) dual-luciferase gene analysis confirmed the target binding of miR-224-5p to GATA3, (c) western blot analysis detected the GATA3 level in the AR cell model, and (d) western blot for detecting the GATA3 level in mice with AR.  ^*∗∗∗*^*P* < 0.001 vs. the NC group or NC mimic group; ^$$^*P* < 0.01, ^$$$^*P* < 0.001 vs. the AR group.

To assess the mechanism by which miR-224-5p is involved in the regulation of AR progression, it was predicted that miR-224-5p targets and binds with GATA3 by the starBase tool (Figure 3(a)). Dual-luciferase gene analysis showed that miR-224-5p observably moderated the luciferase activity of the GATA3 WT group compared to the control group, while there was no prominent alteration in the luciferase activity of the GATA3 MUT group, which confirmed that miR-224-5p targeted GATA3 (Figure 3(b)). In addition, miR-224-5p was overexpressed in the AR cell model and mice with AR, and the role of miR-224-5p in GATA3 expression was analyzed by western blotting. The results showed that GATA3 levels were observably reduced after overexpression of the miR-224-5p mimic in the AR cell model (Figures 3(c) and 3(d)). The above results indicated that miR-224-5p targets GATA3 for negative regulation.

### 3.4. miR-224-5p Inhibits GATA3-Mediated Differentiation of Th1 and Th2 Cells

**Figure 4:**
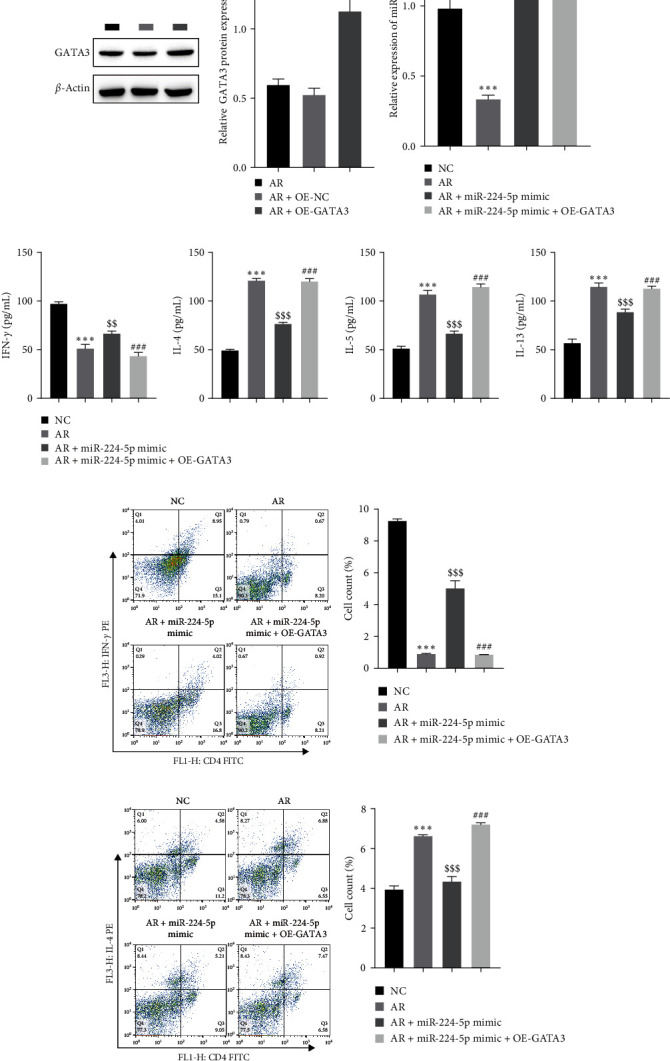
miR-224-5p restrains GATA3-mediated differentiation of Th1 and Th2 cells: (a) western blot analysis for testing the transfection efficiency of GATA3, (b) RT-qPCR for testing the miR-224-5p level, (c) ELISA for testing the levels of IFN-*γ*, IL-4, IL-5, and IL-13, (d) flow cytometry to determine Th1 differentiation, and (e) flow cytometry to detect Th2 differentiation.  ^*∗∗*^*P* < 0.01,  ^*∗∗∗*^*P* < 0.001 vs. the NC group or AR + OE-NC group; ^$$^*P* < 0.01, ^$$$^*P* < 0.001 vs. the AR group; ^###^*P* < 0.001 vs. the AR + miR-224-5p mimic group.

Inspired by the above results, miR-224-5p and GATA3 were overexpressed in CD4 T cells and then cocultured with AR cells to determine the effect of miR-224-5p and GATA3 on CD4 T-cell differentiation. First, western blotting showed successful overexpression of GATA3 (Figure 4(a)). A significant increase in miR-224-5p levels was identified by RT-qPCR after transfection of the miR-224-5p mimic (Figure 4(b)). ELISA showed that IFN-*γ* was increased, and the contents of IL-4, IL-5, and IL-13 were decreased after overexpressing miR-224-5p. The content of IFN-*γ* decreased observably, and IL-4, IL-5, and IL-13 were observably elevated after simultaneous overexpression of miR-224-5p and GATA3 (Figure 4(c)). In addition, Th1 cell differentiation was markedly elevated after overexpressing miR-224-5p and observably decreased after overexpressing miR-224-5p and GATA3 (Figure 4(d)). In contrast, the number of Th2 cells was observably decreased after overexpressing miR-224-5p and observably elevated after overexpressing miR-224-5p and GATA3 (Figure 4(e)). These results indicate that miR-224-5p facilitates Th1 differentiation and restrains Th2 differentiation by restraining GATA3 expression, thus affecting the AR process.

### 3.5. miR-224-5p Attenuates AR Symptoms through GATA3 Regulation of Th1/Th2 Responses

**Figure 5:**
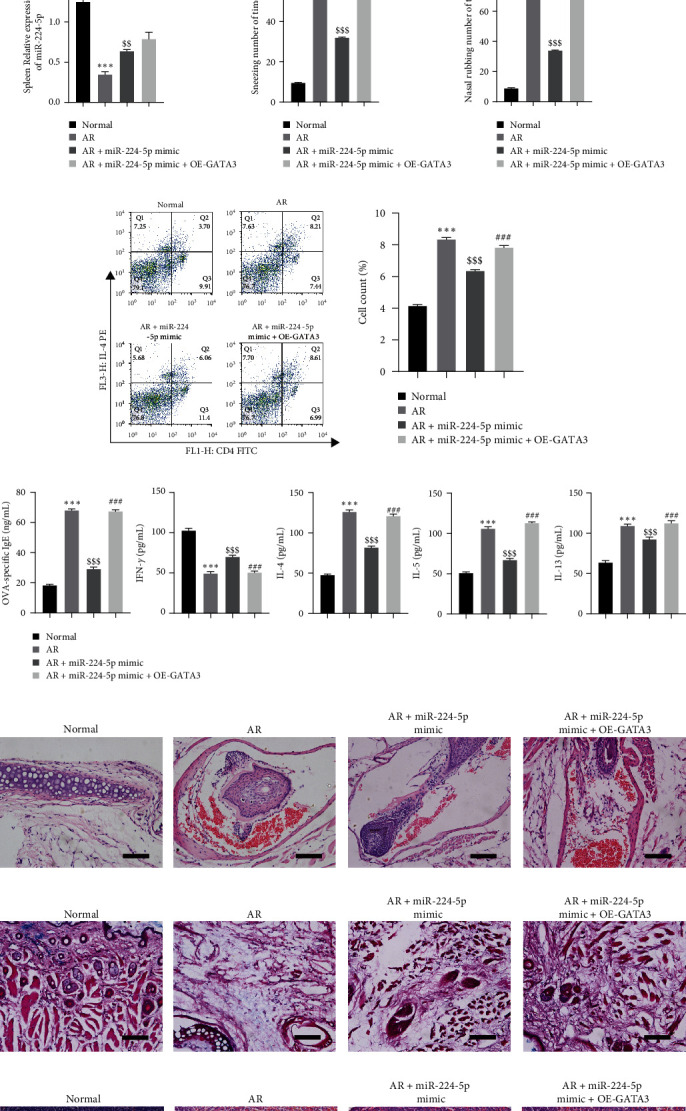
miR-224-5p moderates AR symptoms through GATA3 regulation of Th1/Th2 responses: (a) western blot or testing the GATA3n level in mouse nasal mucosa, (b) RT-qPCR for testing the miR-224-5p level in mouse nasal mucosa, (c) RT-qPCR for testing the miR-224-5p level in mouse spleen, (d, e) observation of the frequency of sneezing and nasal friction, (f) flow cytometry for testing the Th2 differentiation in mouse spleen, (g) ELISA for testing the levels of OVA-specific IgE, IFN-*γ*, IL-4, IL-5, and IL-13 in mouse serum; (h, i) HE and PAS staining for observing histopathological alterations of mouse nasal mucosa (scale bar = 100 *µ*m), and (j) HE staining for observing the histopathological alterations of spleen (scale bar = 100 *µ*m).  ^*∗∗∗*^*P* < 0.001 vs. the normal group; ^$$^*P* < 0.01, ^$$$^*P* < 0.001 vs. the AR group; ^###^*P* < 0.001 vs. the AR + miR-224-5p mimic group.

In mice, miR-224-5p and GATA3 were overexpressed to explore the influence of miR-224-5p on GATA3 expression in AR. GATA3 was observably restrained after overexpressing miR-224-5p in the mouse nasal mucosa, while the GATA3 level was observably elevated after simultaneously overexpressing miR-224-5p and GATA3 (Figure 5(a)). Overexpression of miR-224-5p prominently increased the miR-224-5p level in the nasal mucosa and spleen, as detected by RT-qPCR (Figures 5(b) and 5(c)). The frequency of sneezing and nasal friction in mice was observably lessened after overexpressing miR-224-5p and observably elevated after simultaneously overexpressing miR-224-5p and GATA3 (Figures 5(d) and 5(e)). Overexpression of miR-224-5p restrained Th2 cell differentiation, while overexpression of GATA3 observably elevated Th2 differentiation (Figure 5(f)). Overexpression of miR-224-5p restrained the levels of OVA-specific IgE, IL-4, IL-5, and IL-13 and promoted IFN-*γ* expression, while overexpression of miR-224-5p and GATA3 showed the opposite results compared with the AR group (Figure 5(g)). The results of HE and PAS staining showed that overexpression of miR-224-5p improved eosinophilia and goblet cell hyperplasia, while overexpression of miR-224-5p and GATA3 reversed the above results (Figures 5(h) and 5(i)). HE staining showed that edema and lymphocyte proliferation were observably alleviated after overexpressing miR-224-5p, while edema and lymphocyte proliferation were prominently aggravated after overexpressing miR-224-5p and GATA3 in the spleen (Figure 5(j)). The consequences indicated that miR-224-5p regulates the Th1/Th2 response and inhibits AR inflammation, allergic response, and other symptoms to alleviate AR progression through GATA3.

## 4. Discussion

AR has become a global health problem, although it does not directly threaten the lives of patients [26]. The latest studies have shown that the AR pathological process is extremely intricate, incorporating multifarious immune cells, inflammatory mediators, and cytokines [25]. Further elucidation of the mechanism of AR occurrence and progression and the development of latent targets for AR treatment are needed. It was confirmed that miR-224-5p attenuates allergic responses in mice with AR by regulating Th1/Th2 responses and that GATA3 is critical for miR-224-5p-mediated regulation of AR development.

miRNAs have been shown to be joint regulators of AR development [27]. For example, miR-29 suppresses allergic reactions and symptoms in mice with AR [28]. miRNA-345-5p attenuates AR progression through TLR4/NF-*κ*B pathway-mediated inflammation [29]. miR-133b improves allergic inflammation in an AR model [30]. It has been shown that miR-224-5p is involved in the regulation of multifarious inflammatory diseases. For example, miR-224-5p lessens microglial inflammation by regulating NLRP3 to influence the progression of obstructive sleep apnea and type 2 diabetes [31]. In recent studies, miR-224-5p was confirmed to alleviate AR in mice [17]. In this study, miR-224-5p was decreased in OVA-induced AR mouse nasal mucosa and a Der P1-induced AR cell model, and it has an anti-inflammatory effect in AR development by restraining the expression of IgE, IL-4, IL-5, and IL-13 and facilitating IFN-*γ* expression. Moreover, overexpressed miR-224-5p moderated allergic reactions and lessened sneezing and nasal friction events in mice with AR.

Th1/Th2 imbalance has been proven to be a pivotal factor in AR pathogenesis [32]. Abnormal cytokines generated by IgE-mediated immune responses, inflammatory cell diseases, and Th1/Th2 imbalance have been shown to be involved in AR development [33]. Pathogenic memory Th2 cells play a pivotal role in AR pathogenesis. In general, the levels of IgE, IL-4, IL-5, and IL-13 facilitate Th2 responses to reduce the integrity of nasal epithelial cells in AR [34]. Th1 mainly secretes TNF-*α* and IFN-*γ*, and mediates cellular immune response; Th2 mainly secretes IL-4, IL-5, and IL-10, and mediates humoral immune response. Studies have shown that the two responses are antagonistic. Th1 cytokines (e.g., IFN-*γ*) inhibit the Th2 response, and Th2 cytokines restrain the Th1 response [35]. Hence, it is important to identify the key molecules regulating the Th1/Th2 balance in AR therapy.

The GATA3 transcription factor has been shown to be a prime regulator of Th2 differentiation. GATA3 mediates the expression and activation of IL-4, IL-5, IL-13, and other cytokines to condition Th2 cell differentiation [36]. It has been reported that T-bet, a transcription factor mediating Th1 cell differentiation, can combine with GATA3 to suppress Th2 cell differentiation [37]. In recent years, numerous studies have shown that GATA3 levels can be regulated by miRNAs. For instance, Chen et al. [21] certified that miR-466a-3p targets GATA3 to alleviate allergic nasal inflammation; miR-24 and miR-27 cooperate to restrain Th2 reactions by targeting IL-4 and GATA3 [38]. MiR-224-5p immediately targets GATA3, as predicted by starBase. Moreover, it was found that overexpressed miR-224-5p restrained GATA3 levels, which facilitated CD4 T-cell differentiation into Th1 cells and restrained CD4 T-cell differentiation into Th2 cells in the AR cell model. miR-224-5p regulates the Th1/Th2 response by negatively regulating GATA3, which alleviates eosinophil infiltration and goblet cell proliferation in the nasal mucosa and improves edema and lymphocyte proliferation in the spleen in mice with AR.

## 5. Conclusion

miR-224-5p attenuates allergic reactions in mice with AR by regulating the Th1/Th2 response, and this effect is mediated through GATA3. Overexpressed miR-224-5p can attenuate allergic reactions and symptoms in mice with AR, and miR-224-5p can serve as a latent remedial target for AR progression. Moreover, our findings provide an academic reference for elucidating the pathogenesis of AR.

## Figures and Tables

**Table 1:** Primer sequences.

Genes	Forward primer (5′−3′)	Reverse primer (5′−3′)
miR-224-5p	GCGCGCAAGTCACTAGTGGT	AGTGCAGGGTCCGAGGTATT
U6	CGATACAGAGAAGATTACATGGC	AACGCTTCACGAATTTGCGT

## Data Availability

The datasets used and/or analyzed during the current study are available from the corresponding author upon reasonable request.
